# Population Pharmacokinetics in Oncology and Its Clinical Applications

**DOI:** 10.3390/pharmaceutics16060711

**Published:** 2024-05-25

**Authors:** Nicolas Widmer, Monia Guidi, Thierry Buclin

**Affiliations:** 1 Service of Clinical Pharmacology, Lausanne University Hospital and University of Lausanne, 1011 Lausanne, Switzerland; monia.guidi@chuv.ch (M.G.); thierry.buclin@chuv.ch (T.B.); 2 Pharmacy of the Eastern Vaud Hospitals, 1847 Rennaz, Switzerland; 3 Institute of Pharmaceutical Sciences of Western Switzerland, University of Geneva, University of Lausanne, 1205 Geneva, Switzerland; 4 Center for Research and Innovation in Clinical Pharmaceutical Sciences, Lausanne University Hospital and University of Lausanne, 1011 Lausanne, Switzerland

Most traditional cytotoxic drugs are characterized by steep dose–response relationships and narrow therapeutic windows. Their inter-individual pharmacokinetic (PK) variability is often substantial, making standard dosages produce very different circulating concentration profiles between patients [1,2]. In recent decades, rationally designed small-molecule targeted anticancer drugs—namely, protein kinase inhibitors (PKIs)—have brought considerable progress in therapeutics [3]. They are currently the mainstay of systemic treatment for several hematological malignancies and metastatic solid tumors. They are being increasingly prescribed based on biomarkers predictive of therapeutic response identified through the genetic profiling of tumors, an approach known as precision oncology [4]. Owing to their oral administration route, they offer greater autonomy and simpler outpatient care than cytotoxic chemotherapies and monoclonal antibodies, thus improving patients’ quality of life. However, the therapeutic response to oral targeted drugs varies widely between patients, despite tumor genetic profiling, with insufficient efficacy in certain cases and unacceptable toxicities in others [5]. One of the main causes of this heterogeneity is their PK variability, alongside fluctuating medication adherence, and constitutive or acquired cancer cell drug resistance (i.e., pharmacodynamic [PD] variability) [6]. Finally, PK variability appears to affect, to a clinically significant degree, monoclonal antibodies which are also part of the anticancer armamentarium, such as checkpoint inhibitors [7].

Hence, it is increasingly recognized that definite improvements in the usage of anticancer drugs could be achieved by a better understanding of their PK-PD characteristics and their associated variability, as well as the individualization of their dosage regimen. It is not unusual for the clinical development of new therapeutic agents to result in less-than-optimal dosage recommendations at the time of market entry [8]. We firmly advocate that both qualitative and quantitative aspects of cancer treatment must be considered as part of the precision oncology concept: it encompasses not only the selection of the best anti-cancer agents on the basis of tumor genetic biomarkers, but also the precise individualization of their dosage in each patient. Dosage individualization should be based on both the thorough adaptation to influential patient characteristics (a priori dosage adjustment) and the measurement of plasma circulating drug concentrations (a posteriori adjustment, i.e., therapeutic drug monitoring; TDM) [9,10,11]. These quantitative issues are the focus of pharmacometrics, a discipline that has undergone a remarkable evolution over the last few decades, but of which oncology and cancer patients have yet to take full advantage of. Awareness of this backlog has led the U.S. Food and Drug Administration to initiate Project Optimus, which aims to reform the dose determination paradigm for oncology drugs by relying increasingly on model-informed drug development [12]. State-of-the-art PK and PD analyses of observations gathered during pre-marketing trials (phase II–III) and post-marketing studies (phase IV) are now considered essential to define precise a priori dosage adjustment recommendations. It is highly desirable that they also be exploited extensively to delineate the potential benefit of a TDM strategy, which will require further clinical validation in prospective clinical trials when appropriate.

In that context, this Special Issue presents experimental results and theoretical elaborations that remarkably illustrate the current advances in the PK-PD characterization of anticancer agents, encompassing traditional chemotherapies and novel PKIs.

First, the observational study by Minot-This et al. defined a specific threshold of pazopanib through concentrations (C_min_) associated with better progression-free survival in soft tissue sarcoma (STS) patients. A C_min_ below 27 mg/L was associated with a risk of progression at 3 months. On the other hand, a higher C_min_ in the first 3 months was associated with an increased risk of grade 3–4 toxicities. Thus, pharmacokinetically guided dosing could be helpful to optimize the clinical management of STS patients.

For their part, Courlet et al. investigated the PK-PD relationships for toxicity (absolute neutrophil count) and efficacy (progression-free survival; PFS) using a semi-mechanistic PK-PD model in metastatic breast cancer patients receiving palbociclib. The Cox analyses showed a trend toward a better PFS with increasing palbociclib exposure in older patients. Moreover, model-based simulations confirmed the concentration-dependent and non-cumulative nature of palbociclib-induced neutropenia, and suggested strategies for rational treatment optimization.

In their study, Rodier et al. retrospectively evaluated the exposure–response relationship for the toxicity and efficacy of osimertinib in unselected patients with advanced epidermal growth factor receptor (EGFR)-mutant non-small-cell lung cancer (NSCLC). No significant association between the occurrence of dose-limiting toxicity and plasma exposure was observed in the overall cohort, but the median overall survival (OS) was approximately two-fold shorter with osimertinib at a plasma concentration above 235 ng/mL than at lower concentrations. Similar results were obtained in a cohort of NSCLC patients treated with erlotinib, with the exposure group above 1728 ng/mL showing a six-fold shorter median OS than the lower-concentration exposure groups. These unexpected findings of worsened survival in patients with a high plasma exposure to EGFR inhibitors support the need to abandon the maximum tolerated dose paradigm in the development of PKI dosages.

Regarding cytotoxic drugs, Cauvin et al. used nine different machine learning algorithms to analyze exposure–effect relationships in patients with head and neck cancers treated with a standard cisplatin-based regimen. The generalized linear model was the best algorithm. The peak concentration (C_max_), which ranged between 2.4 and 4.1 µg/mL, was identified as the best exposure marker. Compared with the standard dosage in a series of new patients, the developed Bayesian adaptative dosage strategy would have led to a dose reduction in patients who ultimately proved to have severe toxicities, while increasing dosage in patients with progressive disease.

Taken together, the above-mentioned PK-PD observations illustrate the significance of developing evidence-based TDM through prospective randomized controlled trials. Johnson-Ansah et al. conducted precisely such a trial to assess the value of TDM in chronic myeloid leukemia patients receiving imatinib, starting at 400 mg daily as the first-line therapy (OPTIM-imatinib trial). The patients considered to be underdosed (C_min_ < 1000 ng/mL) were randomized to either a dose escalation strategy aiming to reach the 1000 ng/mL threshold (TDM arm) or the standard imatinib management (control arm). Patients included in the TDM arm had a major molecular response rate of 67% at 12 months compared to 39% in the control arm. This early benefit was maintained over the 3-year study period. Therefore, this study demonstrated that imatinib TDM is feasible and significantly improves response to treatment, confirming previous hints [13].

Moreover, regarding imatinib, Goutelle et al. developed a novel model-informed precision dosing (MIPD) approach and compared its performance with that of other dosing methods. Three target interval dosing (TID) methods were developed based on a previously published PK model to optimize the achievement of a target C_min_ interval and minimize underexposure. The performance of these methods was then compared with that of traditional model-based target concentration dosing (TCD). Both the TID and TCD model-based approaches were effective in simulated and real patients, with 65% and 75%, respectively, achieving the target imatinib exposure (C_min_ between 1000 and 2000 ng/mL). Conversely, the standard 400 mg/24 h dosage of imatinib was associated with less than one-third of target attainment in both real and simulated patients. Combined with subsequent TDM, model-based, goal-oriented methods provide a strong rationale for the precision dosing of imatinib.

Moving a step forward, Goutelle et al. demonstrated the ability to implement and cross-validate a previous population PK model in a new TDM software (Tucuxi, https://www.tucuxi.ch/) for busulfan precision dosing in children undergoing hematopoietic stem cell transplantation. In a subset of patients, the predictive performance of this model in Tucuxi and a reference model in the BestDose software was comparable. Good agreement was reported between the areas under the curve (AUC) and doses estimated by the two MIPDs. Indeed, the pharmacological interpretation of TDM results through MIPD benefits from the development of computer tools of increasing usability and performance [14].

Finally, it is worth noting that new developments are being made to enable point-of-care (POC) TDM at the bedside of cancer patients, as described by Briki et al. in their review article. However, real-time dose adjustment of chemotherapies performed with such POC TDM is only achievable with analytical methods that match the sensitivity and selectivity of the current methods, such as chromatography, as well as MIPD platforms to assist the oncologist with dose fine-tuning based on quantification results and target intervals.

The pharmaceutical industry’s reluctance to embrace any evolution that complicates prescribing hinders the development and dissemination of TDM recommendations that would benefit both patients and the healthcare system. However, rational development lines for the TDM of anticancer agents have been devised and applied by independent researchers [14]. Criteria were developed to prioritize the application of TDM to the therapeutic agents that require it the most [15]. Cancer patients and healthcare providers deserve large-scale efforts to optimize and individualize not only the choice, but also the dosage of anticancer drugs in the context of the widely advocated precision oncology. Prospective randomized controlled trials to assess the level of evidence for the TDM of new PKIs are especially warranted. The development of high-performance, affordable, miniaturized analytical equipment coupled with intelligent, user-friendly MIPD software is underway and very promising. Such systems will assist clinicians in their TDM practice and are expected to disseminate widely [5,16]. Thus, translational efforts in bringing pharmacometrics knowledge closer to medical applications are encouraged, as is the case with the significant research presented in this Special Issue.
